# Endovascular treatment for distal basilar artery occlusion stroke

**DOI:** 10.3389/fneur.2022.931507

**Published:** 2022-08-09

**Authors:** Jiaxing Song, Zhou Yu, Jian Wang, Xiaojun Luo, Jie Du, Zhengxuan Tian, Shunyu Yang, Weihua Xie, Yuqi Peng, Jinlin Mu, Wenjie Zi, Shuchun Huang, Mei Yang

**Affiliations:** ^1^Department of Neurology, Xinqiao Hospital and The Second Affiliated Hospital, Army Medical University (Third Military Medical University), Chongqing, China; ^2^Department of Neurology, The First People's Hospital of Liangshan Yi Autonomous Prefecture, Sichuan, China; ^3^Department of Neurology, Ya'an People's Hospital, Sichuan, China; ^4^Department of Cerebrovascular Diseases, Guangyuan Central Hospital, Sichuan, China; ^5^Department of Neurology, Kaizhou District People's Hospital, Sichuan, China; ^6^Department of Neurology, The 404th Hospital of Mianyang, Sichuan, China; ^7^Department of Neurology, The First People's Hospital of Yunnan, Yunnan, China; ^8^Department of Neurology, People's Hospital of Mengzi, Yunnan, China; ^9^Department of Neurology, Sichuan Science City Hospital, Sichuan, China; ^10^Department of Neurology, Nanjiang County Traditional Chinese Medicine Hospital, Sichuan, China; ^11^Department of Neurology, Hospital 302 Attached to Guizhou Aviation Group, Guizhou, China; ^12^Department of Neurology, Dali Bai Autonomous Prefecture People's Hospital, Yunnan, China

**Keywords:** stroke, distal basilar artery occlusion, endovascular treatment (EVT), occlusion site, acute basilar artery occlusion

## Abstract

**Background:**

This study aimed to investigate the clinical outcomes of endovascular treatment (EVT) for distal basilar artery occlusion (BAO) and compare them with the outcomes of standard medical treatment (SMT) in daily clinical practice.

**Methods:**

Patients with distal BAO enrolled in the BASILAR study from January 2014 to May 2019 were included. Differences in clinical outcomes were analyzed using Pearson's chi-square test and multivariable logistic regression. Clinical outcomes were evaluated using the modified Rankin Scale (mRS) score at 90 days, the mortality at 90 days, and the occurrence of symptomatic intracranial hemorrhage within 48 h.

**Results:**

Among the 267 patients with distal BAO (222 patients in the EVT group and 45 patients in the SMT group), compared with the SMT group, the EVT group was associated with a favorable outcome (mRS 0–3; 40.1 vs. 15.6%; aOR 5.44; 95% CI, 1.68–17.66; *P* = 0.005) and decreased mortality (44.6 vs. 71.1%, aOR 0.32, 95% CI, 0.13–0.77; *P* = 0.012). In the EVT group, multivariable analysis showed that the initial National Institutes of Health Stroke Scale (NIHSS) score and posterior circulation-Alberta Stroke Program Early CT Score (pc-ASPECTS) were associated with favorable functional outcomes and mortality.

**Conclusion:**

Our study suggests that, compared with SMT, EVT is technically feasible and safe for patients with distal BAO.

## Introduction

Acute basilar artery occlusion (BAO) accounts for approximately 1% of all acute ischemic strokes (1, 2). Although the acute BAO incidence is low, it is associated with poor outcomes and high fatality rates (3). Endovascular treatment (EVT) is the standard therapy for acute ischemic stroke in the anterior circulation. Recently, therapy decisions regarding BAO were affected by negative results of several randomized controlled trials comparing standard medical therapy with EVT (3, 4). Although the effectiveness of EVT for acute BAO still lacks strong evidence, clinical neurologists are more likely to choose EVT for acute BAO under real-world conditions.

Our previous multicenter, prospective cohort study indicated that EVT significantly improves the prognosis of patients with BAO, relative to standard medical therapy alone (5). In addition, the ATTENTION Registry also indicated that EVT was associated with significantly better functional outcomes (6). However, the vascular anatomy, clinical presentation, and severity of neurological deficits in patients with BAO in that study were different from those in patients with anterior circulation strokes.

For descriptive purposes, BAO is defined as complete blood flow occlusion in the proximal, middle, or distal basilar artery segment (7). Usually, the anatomy, etiology, and collateral status differ by the affected basilar artery segment, and cerebrovascular accidents involving specific territories supplied by the basilar artery may result in characteristic clinical syndromes, notably the locked-in syndrome and top-of-the-basilar syndrome (2, 8). During EVT, distinct therapeutic strategies may be applied to BAO patients with different occlusion sites. Several studies have focused on the impact of different occlusion locations on ischemic strokes in the anterior circulation. However, only a few reported that the occlusion site is a prognostic factor for BAO; these comprised subgroup analyses and case reports (9, 10).

Thus far, the occlusion site of the distal basilar artery has rarely been the focus of research, and systematic studies or randomized clinical trials in patients with distal BAO undergoing EVT are still lacking. In this multicenter cohort study, we aimed to explore the relationship between the distal BAO location and the clinical prognosis in patients with EVT.

## Methods

### Study design

Patients were selected from the BASILAR registry. BASILAR was a nationwide cohort study between January 2014 and May 2019 that included 47 comprehensive stroke centers from 15 provinces in China. All stroke centers were obliged to enroll consecutive patients to avoid selection bias. All participants or their authorized legal representatives provided written informed consent. BASILAR was registered in the Chinese Clinical Trial Registry (https://www.chictr.org.cn; ChiCTR1800014759).

### Patient selection

Inclusion criteria were as follows: (1) age ≥18 years; (2) presentation within 24 h of the estimated time of BAO; (3) BAO confirmed by computed tomographic angiography (CTA), magnetic resonance angiography (MRA), or digital subtraction angiography (DSA); (4) intravenous recombinant tissue plasminogen activator (rt-PA) treatment initiation within 4.5 h or intravenous urokinase within 6.0 h of the estimated time of BAO; (5) occlusion site in the distal basilar artery [i.e., between the top of the basilar artery to the superior cerebellar artery (SCA)]; and (6) ability to provide informed consent. For the EVT group, EVT had to be initiated within 24 h of the estimated time of BAO. The exclusion criteria were as follows: (1) a clinically significant preexisting disability with a modified Rankin scale (mRS) score of >2; (2) neuroimaging evidence of cerebral hemorrhage on presentation; (3) lack of follow-up information on outcomes at 90 days; (4) current pregnancy or lactation; (5) a serious, advanced, or terminal illness; and (6) incomplete baseline imaging and time-metric data.

### Treatment

Patients with distal BAO were divided into two groups, namely, standard medical treatment group (SMT group) and EVT plus SMT group (EVT group). Patients in the SMT group received SMT as described in the guidelines for the management of acute ischemic stroke (antiplatelet drugs, anticoagulation drugs, intravenous thrombolysis therapy, or their combinations). In the EVT group, patients underwent SMT plus EVT, including mechanical thrombectomy therapies (stent retrievers, thrombo-aspiration, balloon angioplasty, intra-arterial thrombolysis, or their combinations).

### Data collection

Baseline characteristics, stroke risk factors, laboratory data, estimated time of BAO, imaging performance, and functional outcomes at 90 days were recorded (Tables 1, 2).

**Table 1:** Baseline characteristics.

**Variables**	**All patients**				**Propensity Score Matching (1:2)**			
	**Overall**	**SMT**	**EVT**	***P*** **-value**	**Overall**	**SMT**	**EVT**	***P*** **-value**
	**(*n* = 267)**	**(*n* = 45)**	**(*n* = 222)**		**(*n* = 124)**	**(*n* = 44)**	**(*n* = 80)**	
Age, year, [median (IQR)]	69.00 (59.00–76.00)	73.00 (61.00–79.00)	68.00 (59.00–76.00)	0.092	71.00 (59.00–77.25)	72.00 (60.50–78.25)	69.50 (58.00–76.00)	0.403
Sex (%), female	96 (36.0)	16 (35.6)	80 (36.0)	1	37 (29.8)	16 (36.4)	21 (26.2)	0.331
NIHSS baseline [median (IQR)]	29.00 (20.00–34.00)	30.00 (18.00–35.00)	28.00 (20.00–34.00)	0.226	30.00 (22.00–34.00)	30.00 (18.00–35.00)	30.00 (23.00–34.00)	0.775
pc–ASPECTS baseline (median (IQR))^a^	8.00 (7.00–9.00)	7.00 (6.00–9.00)	8.00 (7.00–10.00)	0.02	8.00 (6.00–9.00)	7.00 (6.00–9.00)	8.00 (7.00–9.00)	0.152
PC–CS score [median (IQR)]	5.00 (4.00–6.00)	4.00 (4.00–5.00)	5.00 (4.00–6.00)	0.033	4.00 (4.00–6.00)	4.00 (4.00–5.25)	4.50 (4.00–6.00)	0.06
BATMAN score [median (IQR)]	4.00 (3.00–5.00)	3.00 (2.00–5.00)	4.00 (3.00–5.00)	0.037	4.00 (3.00–5.00)	3.00 (2.00–5.00)	4.00 (3.00–5.00)	0.075
Intravenous thrombolysis (%)	66 (24.7)	21 (46.7)	45 (20.3)	<0.001	56 (45.2)	21 (47.7)	35 (43.8)	0.812
Prodrome (%)	85 (31.8)	9 (20.0)	76 (34.2)	0.09	36 (29.0)	9 (20.5)	27 (33.8)	0.176
SBP [median (IQR)]	149.00 (130.50–167.00)	155.00 (137.00–178.00)	147.50 (130.00–164.00)	0.031	154.00 (135.00–173.00)	155.00 (135.75–178.00)	152.50 (135.00–166.00)	0.339
**Medical history**	
Hypertension (%)	176 (65.9)	29 (64.4)	147 (66.2)	0.955	84 (67.7)	29 (65.9)	55 (68.8)	0.902
Hyperlipidemia (%)	77 (28.8)	12 (26.7)	65 (29.3)	0.863	31 (25.0)	12 (27.3)	19 (23.8)	0.828
Diabetes mellitus (%)	54 (20.2)	10 (22.2)	44 (19.8)	0.871	22 (17.7)	10 (22.7)	12 (15.0)	0.405
Smoking (%)	76 (28.5)	7 (15.6)	69 (31.1)	0.054	36 (29.0)	7 (15.9)	29 (36.2)	0.029
Drinking (%)	54 (20.2)	12 (26.7)	42 (18.9)	0.329	26 (21.0)	12 (27.3)	14 (17.5)	0.294
Atrial fibrillation (%)	117 (43.8)	13 (28.9)	104 (46.8)	0.027	53 (42.7)	13 (29.5)	40 (50.0)	0.044
**Time intervals, min, [median (IQR)]**								
Onset to imaging	186.00 (85.50–298.00)	187.00 (94.00–270.00)	185.00 (85.00–306.50)	0.897	170.00 (85.75–267.00)	186.00 (92.00–267.00)	162.00 (84.25–267.50)	0.468
Onset to treatment	227.00 (128.50–350.00)	236.00 (123.00–339.00)	227.00 (129.75–357.50)	0.76	217.00 (128.25–344.00)	227.50 (122.25–341.75)	215.50 (131.25–344.00)	0.507
Onset to puncture	NA	NA	288.00 (197.25–432.00)	NA	NA	NA	266.00 (196.50–392.00)	NA
Onset to recanalization	NA	NA	378.00 (296.75–515.00)	NA	NA	NA	370.00 (275.50–469.00)	NA
Puncture to recanalization	NA	NA	87.00 (59.75–124.50)	NA	NA	NA	82.00 (57.00–104.50)	NA
Reperfusion, mTICI 2b/3 (%)	NA	NA	188 (84.7)	NA	NA	NA	74 (92.5)	NA
**Stroke etiology (%)**								
LAA	78 (29.2)	13 (28.9)	65 (29.3)	0.026	36 (29.0)	13 (29.5)	23 (28.7)	0.109
CE	151 (56.6)	20 (44.4)	131 (59.0)		68 (54.8)	20 (45.5)	48 (60.0)	
Others	38 (14.2)	12 (26.7)	26 (11.7)		20 (16.1)	11 (25.0)	9 (11.2)	

*IQR, interquartile; SD, standard deviation; NA, not applicable; NIHSS, National Institutes of Health Stroke Scale; pc-ASPECTS, posterior circulation-Alberta Stroke Program Early CT Score; PC-CS, posterior circulation collateral score; BATMAN, basilar artery on Tomography Angiography; SBP, systolic blood pressure; LAA, large artery atherosclerosis; CE, cardioembolism; SMT, standard medical treatment*.

a *Data were missing for two patients in the EVT group and one patient in the SMT group*.

**Table 2:** Primary and secondary efficacy outcomes and safety outcomes.

**Characteristic**	**All patients**	**Propensity score matching (1:2)**									
	**Overall** **(*n* = 267)**	**SMT** **(*n* = 45)**	**EVT** **(*n* = 222)**	**Unadjusted OR** **(95% CI)^a^**	***P*** **-value**	**Adjusted OR** **(95% CI)^b^**	***P*** **-value**	**Overall** **(*n* = 124)**	**SMT** **(*n* = 44)**	**EVT** **(*n* = 80)**	***P*** **-value**
**Primary efficacy outcomes–*****n*****/total** ***n*** **(%)**											
mRS 0–3 at 90 days	96 (36.0)	7 (15.6)	89 (40.1)	3.63 (1.55–8.50)	0.003	5.44 (1.68–17.66)	0.005	35 (28.2)	7 (15.9)	28 (35.0)	0.04
**Secondary efficacy outcomes–*****n*****/total** ***n*** **(%)**											
mRS 0–2 at 90 days	82 (30.7)	7 (15.6)	75 (33.8)	2.77 (1.181–6.50)	0.019	3.69 (1.12–12.09)	0.031	30 (24.2)	7 (15.9)	23 (28.7)	0.168
mRS 0–1 at 90 days	58 (21.7)	6 (13.3)	52 (23.4)	1.99 (0.79–4.96)	0.141	1.68 (0.53–5.32)	0.378	23 (18.5)	6 (13.6)	17 (21.2)	0.422
**Safety outcomes–*****n*****/total** ***n*** **(%)**											
Mortality at 90 days	131 (49.0)	32 (71.1)	99 (44.6)	0.33 (0.16–0.66)	0.002	0.32 (0.13–0.77)	0.012	68 (54.8)	31 (70.5)	37 (46.2)	0.016
sICH	15 (5.7)	0 (0.0)	15 (6.9)	2.96 (1.49–5.88)	0.002	NA	0.997	5 (4.0)	0 (0.0)	5 (6.2)	0.224

*mRS, modified Rankin scale; sICH, symptomatic intracranial hemorrhage; EVT, endovascular treatment; NA, not applicable; SMT, standard medical treatment*.

a *The odds ratios were estimated from a binary logistic regression model*.

b *Adjusted estimates of outcome were calculated using multiple regression, taking the following variables into account: age, sex, baseline NIHSS score, baseline pc-ASPECTS, atrial fibrillation, Intravenous Thrombolysis, PC-CS Score, and Stroke etiology*.

The causative mechanism of stroke was assessed based on the Trial of ORG10172 in Acute Stroke Treatment (TOAST) classification (11). The National Institutes of Health Stroke Scale (NIHSS) was used to assess neurological deficits at the time of treatment, with a higher NIHSS score indicating more severe neurological disability. The posterior circulation Alberta Stroke Program Early Computed Tomography Score (pc-ASPECTS; range, 0–10, a lower score indicating more extensive ischemia) was used to quantify ischemic changes on baseline images. The collateral circulation status was assessed using the posterior circulation-collateral score (PC-CS). The modified thrombolysis in cerebral infarction (mTICI), wherein a grade of 2b or 3 indicates successful reperfusion after EVT, was also used. Based on angiographic findings (CTA, MRA, or DSA), we classified the occlusion site into the distal basilar artery (distal to the SCA), middle basilar artery (from the anterior inferior cerebellar artery (AICA) to the SCA), and proximal basilar artery (from the vertebrobasilar junction to the AICA) (7).

### Outcome measures

The primary clinical efficacy outcome was a favorable outcome, defined as an mRS score (range, 0–6, with 0 indicating no disability, 3 indicating moderate disability, and 6 indicating death) of 0–3 at 90 days. The secondary clinical efficacy outcome was the rate of functional outcomes, defined by an mRS score of 0 to 2 or 0 to 1 (indicating an ability to walk unassisted) at 90 days.

Safety outcomes included symptomatic intracerebral hemorrhage (sICH) within 48 h as confirmed by neuroimaging (CT or MRI) and mortality at 90 days. sICH was defined according to the Heidelberg Bleeding Classification (hemorrhagic transformation of infarcted brain tissue, intracerebral hemorrhage both within and outside the infarcted brain tissue, intracerebral hemorrhage outside the infarcted brain tissue, or intracranial extracerebral hemorrhage, and an increase of ≥4 points in the NIHSS score or an increase of ≥2 points in one of the 11 NIHSS subcategories).

### Statistical analysis

We compared baseline characteristics and outcomes between the SMT and EVT groups. Categorical variables were described as proportions and compared using the χ^2^ test or Fisher's exact test; continuous variables were described as mean (SD) or median (IQR) and compared using the *t*-test or equivalent nonparametric tests.

To adjust for confounders, we used a multivariable logistic regression analysis model and entered covariates such as age, sex, atrial fibrillation, IVT, NIHSS score, pc-ASPECTS, collateral status, and stroke etiology. Odds ratios (ORs) were calculated with 95% confidence intervals (CIs) to indicate statistical precision. To generate margin effect curves related to the treatment modalities, outcome-specific predicted probabilities for continuous age values were computed by setting other variables in the model to their mean values. The Kaplan-Meier method and log-rank test were used to analyze the mortality.

For propensity score matching analyses, we performed 1:2 matching based on the nearest-neighbor matching algorithm with a caliper width of 0.2 of the propensity score with age and intravenous thrombolysis as covariates (12). Furthermore, supportive analyses used the propensity score and computed based on multivariable regression models accounting for additional explanatory variables were performed. The significance level was set to *P* < 0.05, and all tests of the hypotheses were two-sided. Since we excluded patients with missing essential data from our analysis, we did not impute for missing data. Statistical analyses were performed using SPSS (version 26.0; IBM, Armonk, NY, USA) and R version 4.1.0 (R packages: tableone, MatchIt, ggeffects, forestplot, ggplot2, survminer, R Foundation for Statistical Computing, Vienna, Austria).

## Results

### Baseline characteristics

In total, 829 patients with acute ischemic stroke at our study between January 2014 and May 2019 were included. The proximal basilar artery was affected in 121 patients, the middle basilar artery in 295 patients, and the vertebral artery-V4 segment in 146 patients. Therefore, the distal BAO cohort comprised 267 patients, with 45 and 222 patients in the SMT and EVT groups, respectively (Supplementary Figure I).

The baseline characteristics are summarized in Table 1. The median age was 69 years (IQR, 59–76), and 96 patients (36.0%) were women. The median NIHSS score was 29 (IQR, 20–34), with no between-group difference. Patients in the EVT group had a pc-ASPECTS [7 (IQR, 6–9) vs. 8 (IQR, 7–10); *P* = 0.02]. There was no imbalance in time intervals between groups. Other baseline characteristics were balanced between groups.

### Outcome of EVT vs. SMT in distal BAO

**Figure 1:**
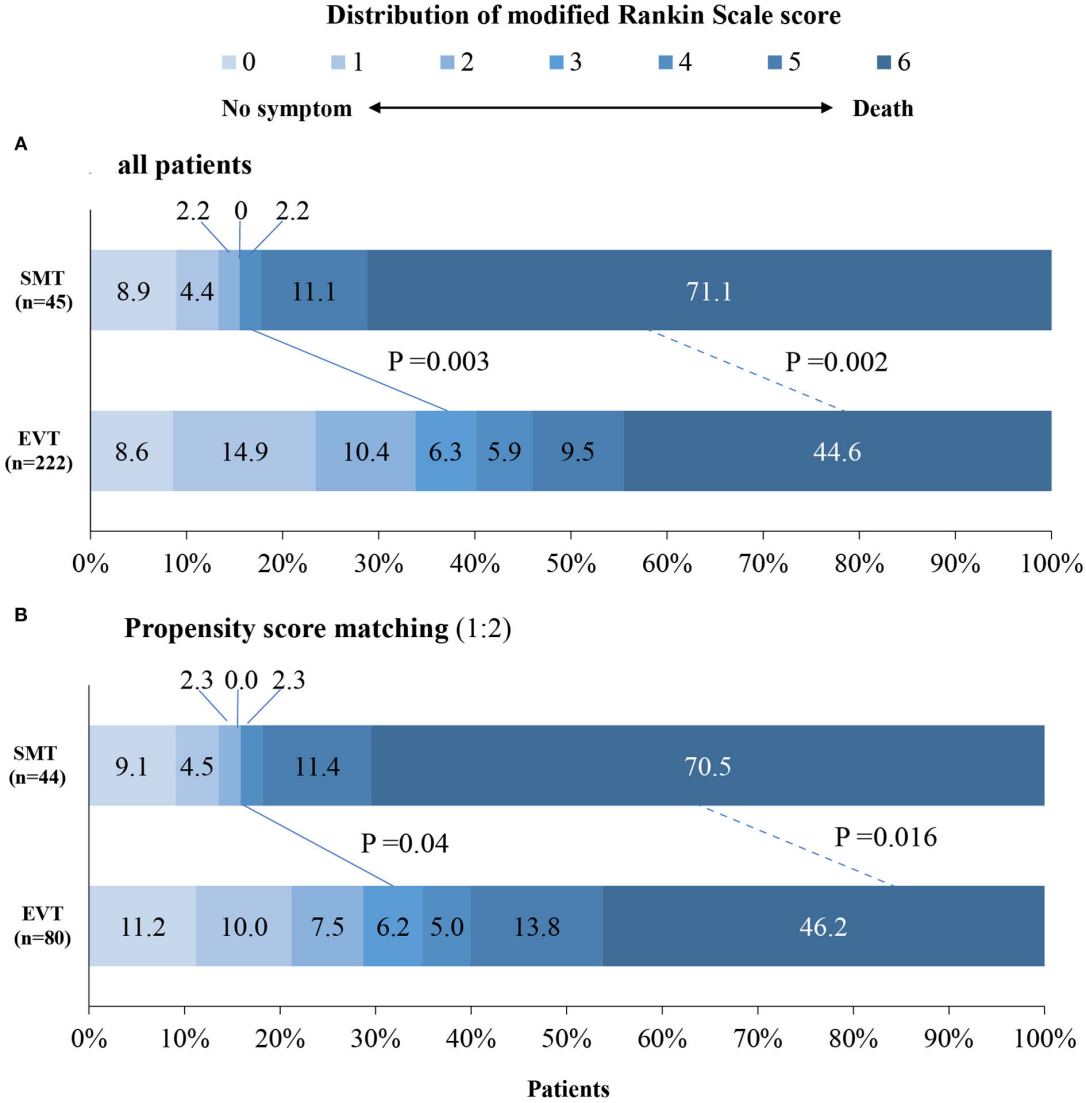
Distribution of modified Rankin scale scores at day 90 in patients with distal BAO. The distribution of the modified Rankin scale scores at day 90 in patients with distal BAO is shown. Clinical outcomes at day 90 follow-up in patients in the SMT group vs. those of the EVT group **(A)** and clinical outcomes at day 90 follow-up after propensity score matching **(B)**. BAO, basilar artery occlusion; EVT, endovascular treatment; SMT, standard medical treatment.

**Figure 2:**
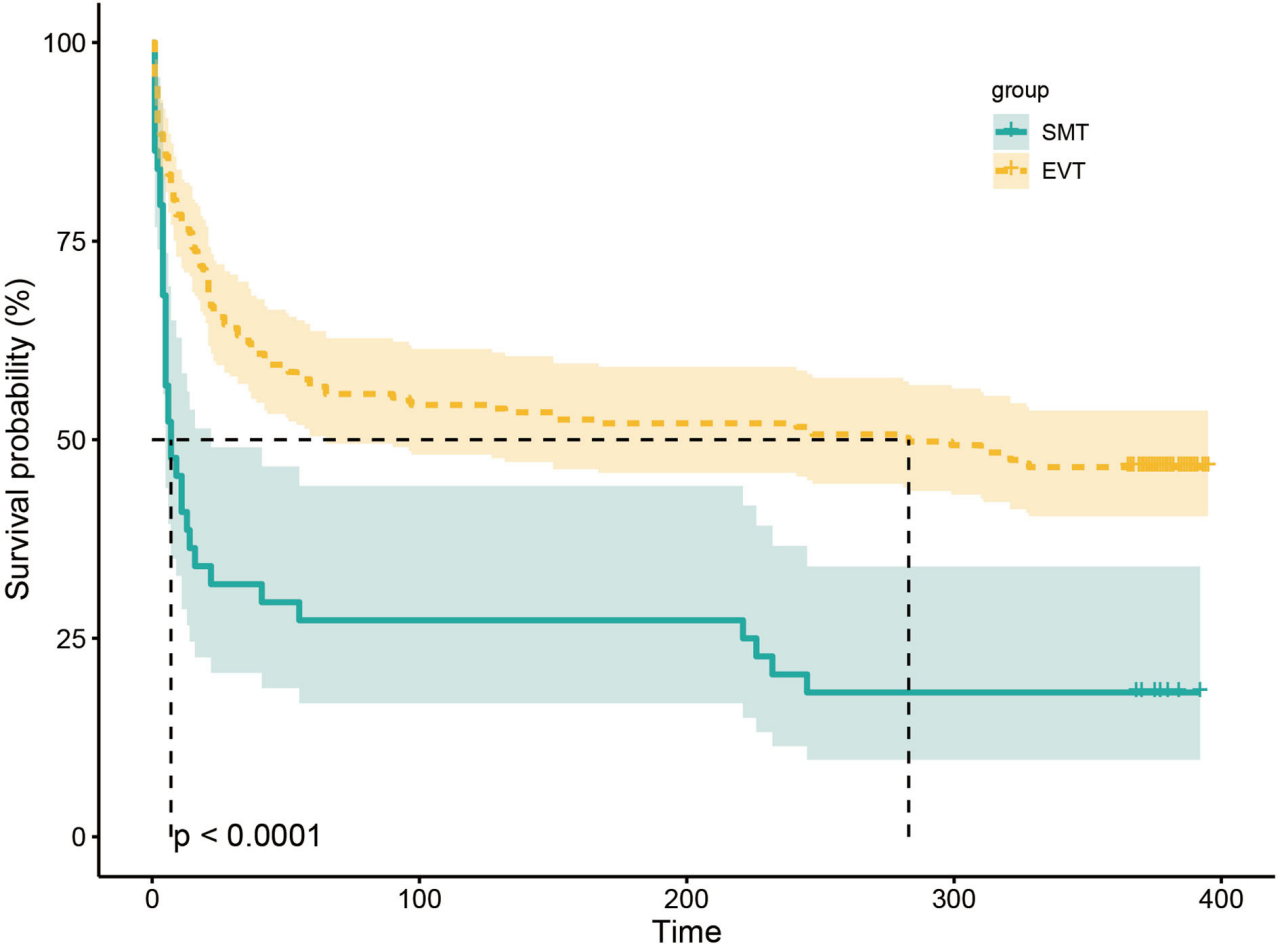
Kaplan–Meier curve estimates of the probability of death during the 1-year follow-up. The probability of death during the 1-year follow-up in SMT and EVT groups. SMT, standard medical treatment; EVT, endovascular treatment.

**Figure 3:**
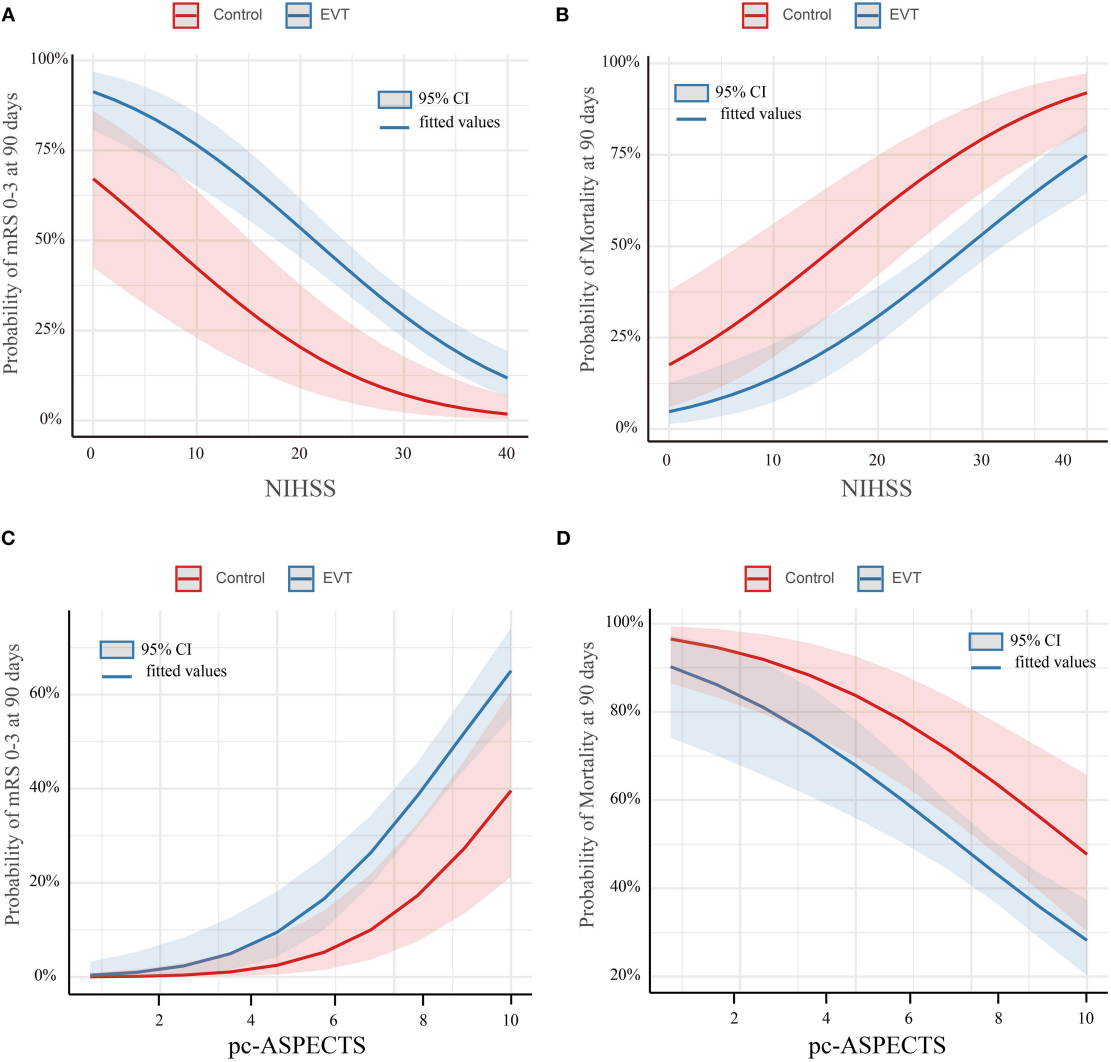
Predicted probability of clinical outcomes by NIHSS score and pc-ASPECTS in patients with distal BAO. Predicted probability of a favorable outcome **(A)** and mortality **(B)** by NIHSS in patients with distal BAO and the predicted probability of a favorable outcome **(C)** and mortality **(D)** by pc-ASPECTS in patients with distal BAO. Curves showed that among patients with distal BAO, the EVT group showed a higher predicted rate of favorable functional outcome and a lower predicted rate of mortality than the SMT group. Shading indicates the 95% confidence interval. BAO, basilar artery occlusion; EVT, endovascular treatment; NIHSS, National Institutes of Health Stroke Scale; pc-ASPECTS, posterior circulation-Alberta Stroke Program Early CT Score; SMT, standard medical treatment.

The study outcomes are presented in Figure 1A and Table 2. Figure 1A shows the distribution of mRS scores at 90 days in the two groups. In univariable analysis, 89 of 222 patients (40.1%) in the EVT group and seven of 45 patients (15.6%) in the SMT group had a favorable functional outcome (*P* = 0.003). Mortality at 90 days was 44.6% in the EVT group and 71.1% significantly higher in the SMT group (*P* = 0.002). The rate of symptomatic intracranial hemorrhage was 6.9% in the EVT group but 0% in the SMT group (*P* = 0.002). In addition, there was a significant difference in survival rate between patients with EVT and SMT during the follow-up of 1 year (Figure 2). In the multivariable analysis with adjusted confounders, EVT was found to be significantly positively correlated with favorable functional outcomes (mRS score 0–3 at 90 days; aOR, 5.44; 95% CI, 1.68–17.66; *P* = 0.005). Likewise, EVT was positively correlated with good functional outcomes (mRS score 0–2 at 90 days; aOR, 3.69; 95% CI, 1.12–12.09; *P* = 0.031). Additionally, EVT was found to be negatively correlated with mortality (aOR, 0.32; 95% CI, 0.13–0.77; *P* = 0.012; Table 2). Among patients in both EVT and SMT groups, the NIHSS score and pc-ASPECTS were independently associated with the clinical outcomes. The predicted probability curves of clinical outcomes by NIHSS score and pc-ASPECTS in patients with distal BAO and EVT are shown in Figure 3.

**Figure 4:**
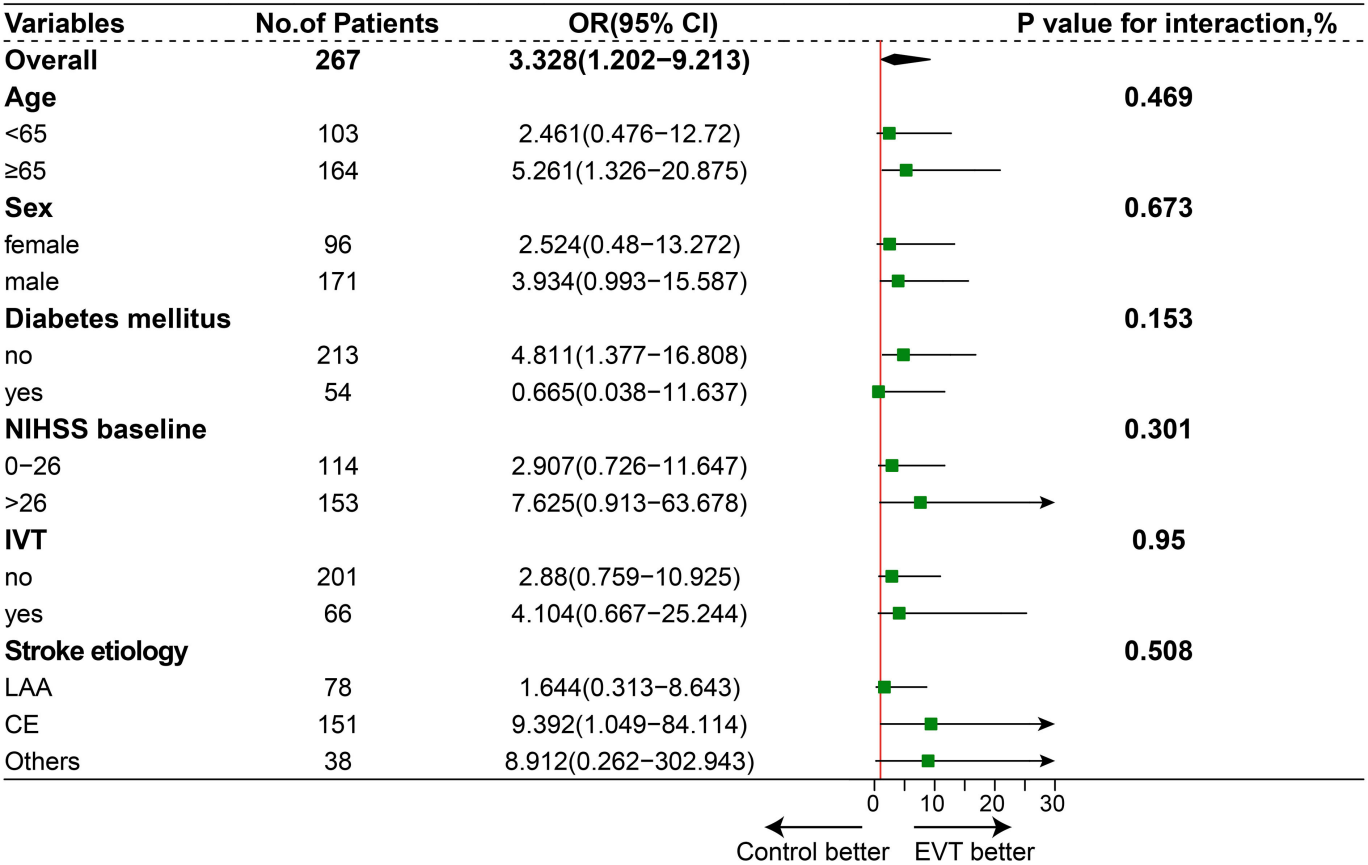
Subgroup analysis. The forest plot shows the difference in odds ratio (OR) for favorable outcomes (defined as a modified Rankin scale score of 0–3) at day 90 in the subgroup. Adjusted variables were age, sex, National Institutes of Health Stroke Scale (NIHSS) score, diabetes mellitus (DM), and intravenous thrombolysis (IVT), stroke etiology.

After 1:1 propensity score matching, the baseline characteristics of the two groups achieved a good balance, and the details are presented in Table 1 and Supplementary Methods. The proportion of favorable functional outcomes was significantly higher in the EVT group (35.0 vs. 15.9%; *P* = 0.04). Mortality at 90 days occurred in 37 of 80 patients (46.2%) in the EVT group and 31 of 44 patients (70.5%) in the SMT group (*P* = 0.016). The rate of symptomatic intracerebral hemorrhage was 6.2% (5 of 80 patients) in the EVT group and 0% in the SMT group (0 of 44 patients; *P* = 0.224). The results are shown in Figure 1B and Table 2. A subgroup analysis was performed to investigate the relationship between the two groups and 90-day functional outcomes (mRS score 0–3). The cutoff values and subgroup categories were based on the median values, including those based on age, sex, NIHSS score, diabetes mellitus, pc-ASPECTS, and intravenous thrombolysis. The results showed no significant heterogeneity across any of the prespecified subgroups (Figure 4).

### Predictors of outcome with EVT in patients with distal BAO

To identify the predictors of outcome in the EVT group, 222 patients with distal BAO were dichotomized by functional outcome (favorable vs. poor; Supplementary Table I) and mortality (living vs. dead) to determine the factors to be adjusted. In the multivariable analysis with adjusted confounders, baseline NIHSS score (aOR, 0.91; 95% CI, 0.87–0.95; *P* < 0.001), baseline pc-ASPECTS (aOR, 1.65; 95% CI, 1.30–2.08; *P* < 0.001), Pneumonia (aOR, 0.38; 95% CI, 0.17–0.85; *P* = 0.019), and PTR (aOR, 0.99; 95% CI, 0.98–1.00; *P* = 0.039) were associated with favorable functional outcome. Additionally, baseline SBP (aOR, 1.01; 95% CI, 1.00–1.03; *P* = 0.036), baseline NIHSS score (aOR, 1.11; 95% CI, 1.06–1.16; *P* < 0.001), baseline pc-ASPECTS (aOR, 0.76; 95% CI, 0.63–0.91; *P* = 0.003), PTR (aOR, 1.01; 95% CI, 1.003–1.017; *P* = 0.039), and successful reperfusion (aOR, 0.29; 95% CI, 0.11–0.78; *P* = 0.014) were associated with mortality (Table 3).

**Table 3:** Multivariable analysis: predictors of clinical outcome (mRS 0–3, mortality) in the EVT group.

**Variables**	**Unadjusted OR**	***P*** **-value**	**Adjusted OR**	***P*** **-value**
	**(95% CI)**		**(95% CI)**	
**Functional outcome**				
NIHSS baseline	0.90 (0.87–0.93)	<0.001	0.91 (0.87–0.95)	<0.001
pc-ASPECTS baseline	1.65 (1.36–2.00)	<0.001	1.65 (1.30–2.08)	<0.001
Pneumonia	0.31 (0.17–0.57)	<0.001	0.38 (0.17–0.85)	0.019
PTR	0.99 (0.98–0.99)	<0.001	0.99 (0.98–1.00)	0.039
Number of attempts	0.75 (0.58–0.98)	0.035	0.93 (0.65–1.33)	0.699
Anesthesia strategy	0.46 (0.26–0.83)	0.009	1.03 (0.47–2.27)	0.934
**Stroke etiology**				
LAA^a^	Reference	0.429	Reference	0.479
CE	0.68 (0.37–1.23)	0.201	0.80 (0.25–2.57)	0.707
Others	0.86 (0.34–2.14)	0.739	0.56 (0.19–1.65)	0.296
**Mortality**				
NIHSS baseline	1.10 (1.06–1.14)	<0.001	1.11 (1.06–1.16)	<0.001
pc-ASPECTS baseline	0.69 (0.59–0.82)	<0.001	0.76 (0.63–0.91)	0.003
PTR	1.01 (1.01–1.02)	<0.001	1.01 (1.003–1.017)	0.003
Diabetes mellitus	1.85 (0.95–3.60)	0.071	2.01 (0.90–4.47)	0.087
Age	1.022 (0.99–1.05)	0.056	1.02 (0.99–1.04)	0.296
Successful reperfusion	0.20 (0.08–0.50)	<0.001	0.29 (0.11–0.78)	0.014
SBP	1.01 (0.99–1.021)	0.072	1.01 (1.00–1.03)	0.036

*OR, odds ratio; CI, confidence interval; NIHSS, National Institutes of Health Stroke Scale; SBP, systolic blood pressure; pc-ASPECTS, posterior circulation-Alberta Stroke Program Early CT Score; EVT, endovascular treatment; OTT, onset-treatment time; LAA, large artery atherosclerosis; CE, cardioembolism; PTR, puncture to recanalization time*.

a *Large artery atherosclerosis was taken as a reference*.

## Discussion

To the best of our knowledge, this study is the first to determine the impact of distal BAO on patient prognosis. We investigated the efficacy and safety of EVT compared with those of SMT in patients with distal BAO. The outcomes were significantly different between patients who received EVT for distal BAO initiated within 24 h after stroke onset and those who received only medical therapy.

The incidence of a favorable outcome in patients with distal BAO was higher in the EVT group than in the SMT group. Mortality and symptomatic intracranial hemorrhage rates did not differ between groups. Moreover, after propensity score matching, the results still indicated that endovascular treatment is technically feasible and safe for distal BAO. In line with our findings, previous studies have reported favorable outcomes for additional EVT in patients with distal BAO (13, 14).

Thus, our study demonstrates the benefit of endovascular treatment for distal BAO. From the perspective of anatomical characteristics, the basilar artery, which is the main vessel of the posterior circulation, supplies most of the brainstem and occipital lobes, as well as parts of the cerebellum and thalamus (2). An ischemic stroke occurring at the top of the basilar artery often causes infarction of the midbrain, thalamus, and portions of the temporal and occipital lobes supplied by the posterior communicating and posterior cerebral branches of the basilar artery. These territories regulate the consciousness level, vision, and other functions (7, 8), and patients with distal BAO can show various clinical signs and symptoms, including consciousness disorder, hemianopia, oculomotor disorders, and behavioral abnormalities rather than prominent motor dysfunction. In addition, the corticospinal tracts of these patients usually remain intact (8, 15). Therefore, patients may achieve better therapeutic outcomes with additional EVT of the distal basilar artery than with SMT alone.

As reported previously, the stroke mechanism has a major influence on favorable outcomes. Similar to ischemic stroke, BAO can be caused by various stroke mechanisms. The two most common mechanisms underlying BAO are cardioembolism and large artery atherosclerosis (16, 17). Some studies have shown that atherosclerosis often leads to occlusion of the proximal and middle segments of the basilar artery, whereas embolic BAO often manifests in the distal segment (2, 18, 19). It is easy to conceive that a cardiac embolus leads to acute BAO, resulting in the sudden onset of neurological deficits. In previous studies, large artery atherosclerosis was found to be more frequent in patients with progressive presentations (2, 17). Thus, in distal BAO caused by emboli, patients often have abrupt courses without premonitory symptoms and are more likely to arrive at the hospital in time; this offers a better therapeutic time window.

Patients with large artery atherosclerosis often have poor clinical outcomes following EVT (18). Patients with atherosclerosis require a longer procedural time than those with emboli (20). Moreover, atherosclerotic lesions, such as plaque rupture, endothelial damage, or irritation, and local platelet activation-prone situations frequently cause acute arterial reocclusion after EVT (21, 22); this reocclusion requires repetitive thrombectomy procedures and additional rescue treatments. In contrast, the proportion of reocclusion in the embolism group is lower than that in the atherosclerosis group due to mild or no arterial wall injury, less damage to perforating branches, and reduced thrombectomy time. Similar to previous findings, our results suggest that patients with distal occlusion have a more favorable outcome following EVT (18, 23, 24).

Recently, a subgroup analysis of the Basilar Artery International Cooperation Study (BASICS) trial showed that EVT has no treatment benefit compared with SMT alone for distal BAO (3). First, in the SMT group, the median NIHSS score was 27 in our study, whereas this score was 22 in the BASICS trial, suggesting that the overall disease severity was lower in the BASICS trial. Second, the proportion of intravenous thrombolysis was 20% in our study and approximately 80% in the BASICS trial. In this case, in addition to antiplatelet or anticoagulant therapy, patients can receive more intravenous thrombolysis therapy, which may result in better clinical efficacy in the medical care group and abolish the differences between the two groups in terms of the prognosis. Third, since recruitment was lesser than anticipated, the BASICS trial was underpowered for some analyses, including this subgroup analysis. Finally, clinical patterns and healthcare policy decision-making differ between developed and developing countries; developing countries have a higher stroke burden and more limited healthcare resources.

### Limitations

Our study has several limitations. First was the nonrandomized design. Second, treatment allocation in the participating centers was based on medical conditions at local hospitals and individual decisions of the treating physicians; therefore, bias cannot be excluded. Moreover, the treatment groups were not balanced. Third, propensity score matching or multivariable analyses could not adjust completely for systematic differences between treatment groups, which is the aim of randomization in clinical trials. Therefore, we suggested that a randomized controlled trial in patients with acute BAO is of high priority. However, our study provides a good representation of daily clinical practice for patients with acute distal BAO, and despite its limitations, it still provides one of the best available datasets regarding the outcomes of distal BAO treatment.

## Conclusion

Our study suggests that, compared with SMT, EVT is technically feasible and safe for distal BAO. In the future, the efficacy of EVT compared with that of SMT for distal BAO needs to be assessed in randomized controlled trials.

## Data availability statement

The raw data supporting the conclusions of this article will be made available by the authors, without undue reservation.

## Ethics statement

The studies involving human participants were reviewed and approved by Xinqiao Hospital Affiliated to Army Medical University. ID: 201308701. The patients/participants provided their written informed consent to participate in this study.

## Author contributions

SH, MY, and JS contributed to the conception and design of this study. JS, ZY, JW, XL, JD, ZT, SY, WX, YP, JM, and WZ contributed to the acquisition and analysis of data. JS and ZY contributed to drafting the text or preparing the figures. All authors critically reviewed and approved the manuscript.

## Funding

The authors disclosed receipt of the following financial support for the research, authorship, and/or publication of this study. This study was supported by the National Natural Science Foundation of China (No. 82071323), the Chongqing Natural Science Foundation (cstc2020jcyj-msxmX0926), the Army Medical University Clinical Medical Research Talent Training Program (2019XLC2008), and the Clinical Medical Research Talents Training Program of Army Military Medical University (2018XLC1005).

## Conflict of interest

The authors declare that the research was conducted in the absence of any commercial or financial relationships that could be construed as a potential conflict of interest.

## Publisher's note

All claims expressed in this article are solely those of the authors and do not necessarily represent those of their affiliated organizations, or those of the publisher, the editors and the reviewers. Any product that may be evaluated in this article, or claim that may be made by its manufacturer, is not guaranteed or endorsed by the publisher.
