# Supplementary material for: Endovascular treatment for distal basilar artery occlusion stroke
Source: Front Neurol. 2022 Aug 9;13:931507. doi: 10.3389/fneur.2022.931507 (PMC9395985; doi:10.3389/fneur.2022.931507)

**Supplementary materials**

Supplemental Methods. Propensity score matching analysis.

Supplemental Table I. Baseline characteristic in distal BAO with EVT.

Supplemental Figure I. Study flowchart.

This supplementary material has been provided by the authors to give readers additional information about their work.

eMethods. Propensity score matching analysis

We performed a 1:1 propensity score matching based on the nearest–neighbor matching algorithm with a caliper width of 0.2 using R (R Core Team. R: A Language and Environment for Statistical Computing. R Foundation for Statistical Computing. Vienna, Austria, 2021). 2 patients in the EVT group and 1 patient in the SMT group were excluded prior to propensity matching due to lack of pc–ASPECTS baseline.

Propensity score matching Variables & Method

Variables:

- Age
- IVT (intravenous thrombolysis)

Matching：

- Method = “nearest”
- distance = “logit”
- ratio = 2
- caliper = .2

| Sample Sizes: | | |
| --- | --- | --- |
|  | Control | Treated |
| All | 44 | 220 |
| Matched | 44 | 80 |
| Unmatched | 0 | 140 |
| Discarded | 0 | 0 |

**Matching Graphs**

Propensity score distribution


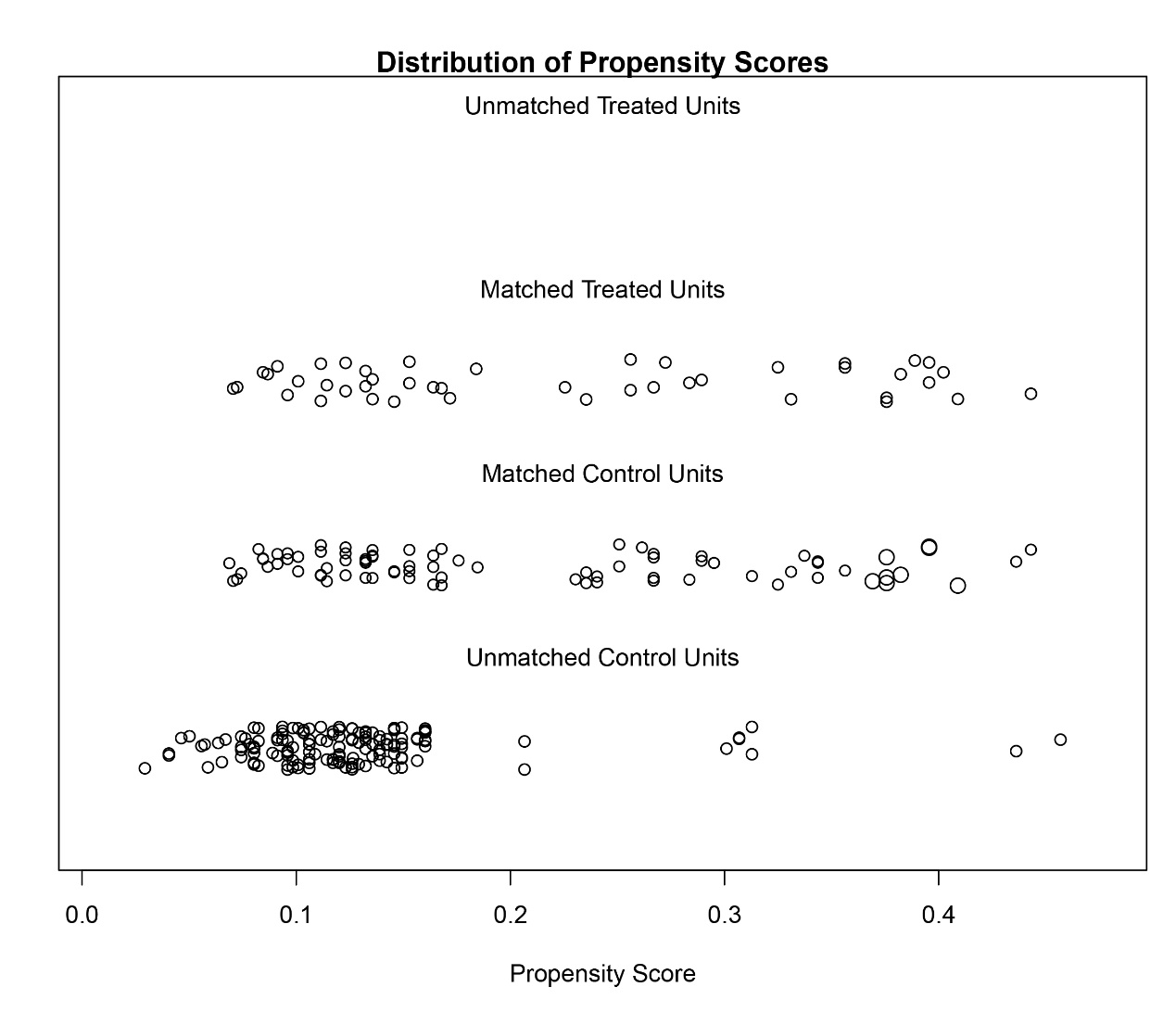


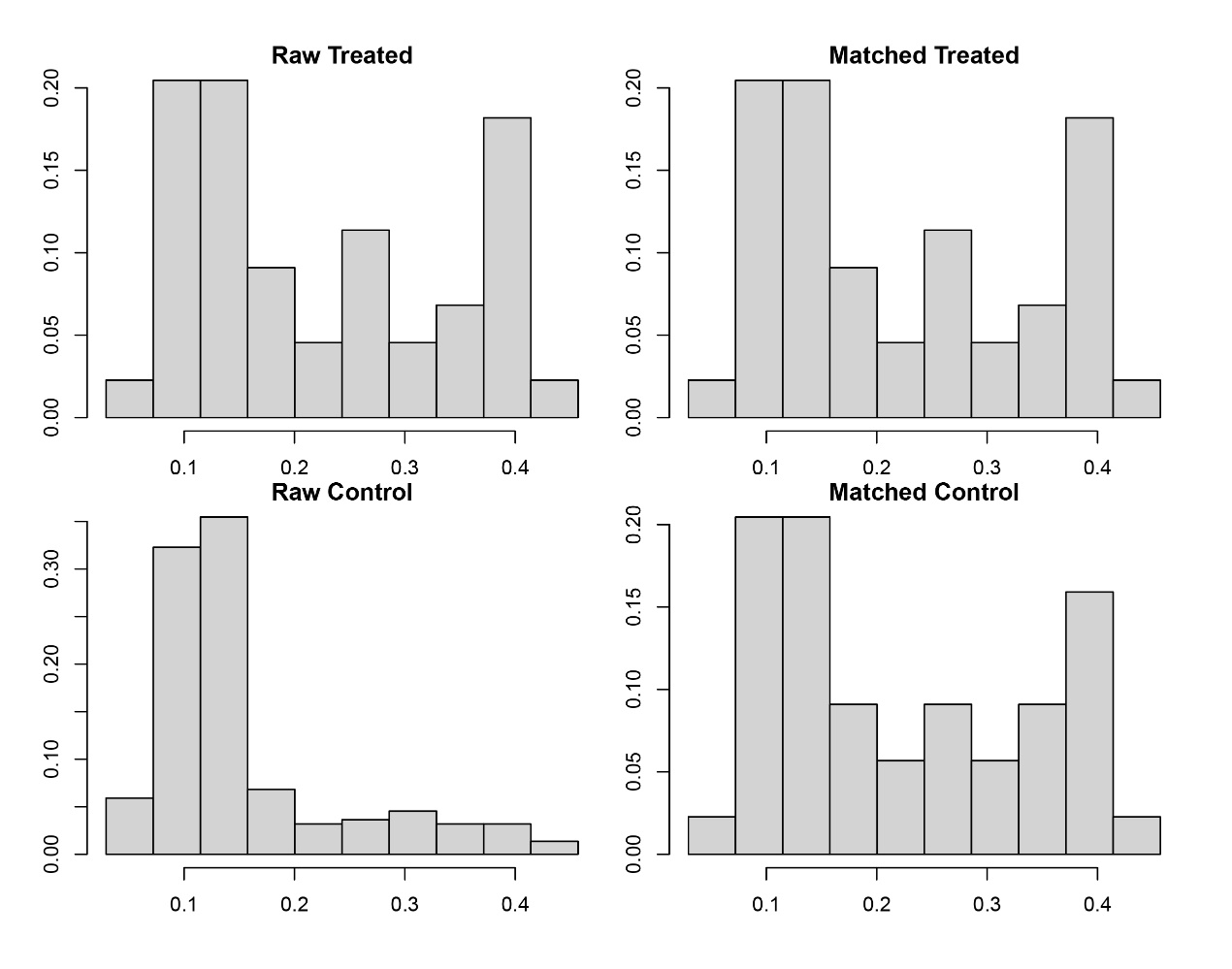


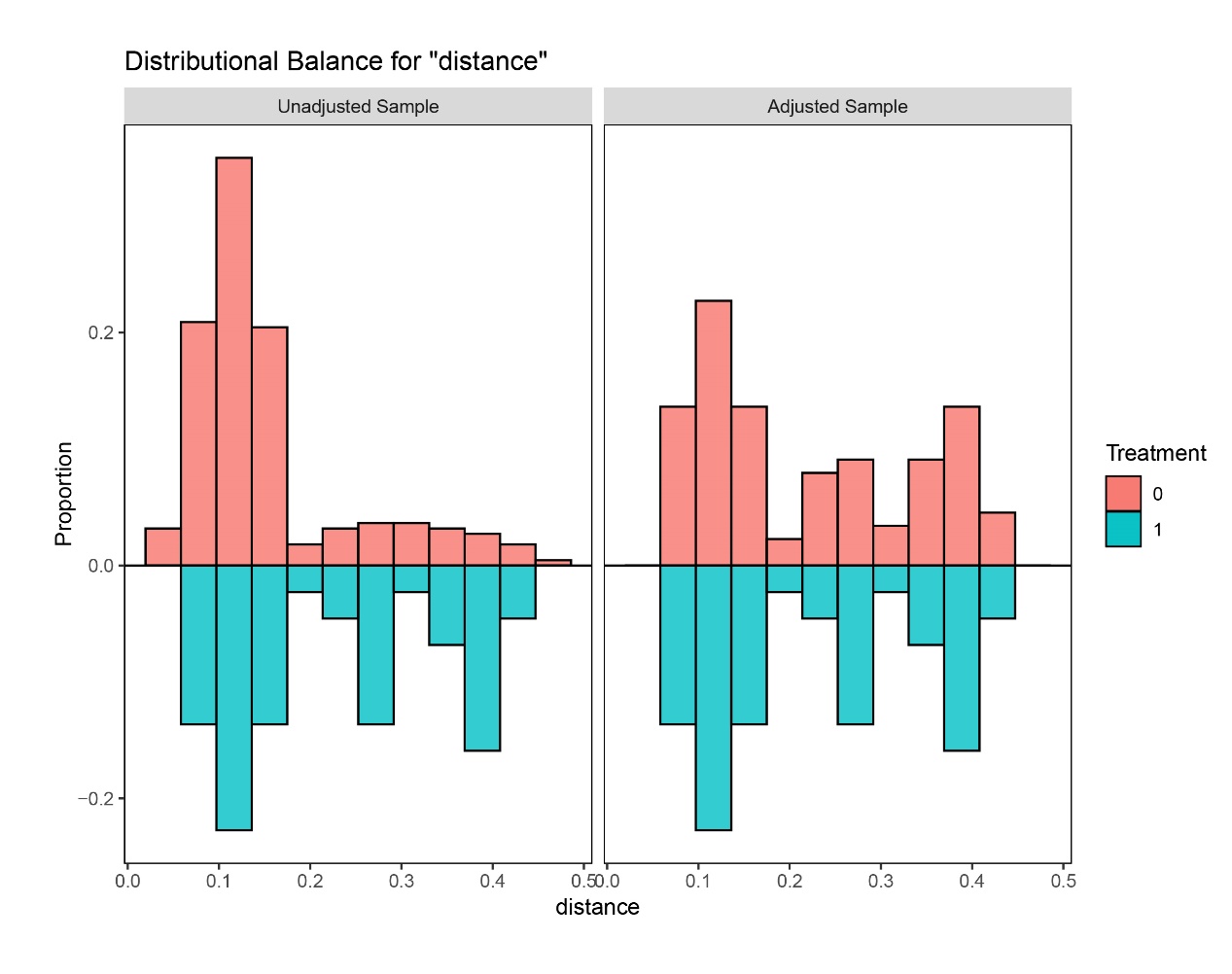


Table Ⅰ. Baseline characteristic in distal BAO with EVT.

| **Variables** | **Overall** | **Poor outcome**  **(mRS 4-6)** | **Good outcome**  **(mRS 0-3)** | ***P* value** |
| --- | --- | --- | --- | --- |
|  | **n=222** | **n=133** | **n=89** |  |
| Age (median (IQR)), y | 68.00 (59.00- 76.00) | 69.00 (61.00- 76.00) | 65.00 (57.00- 75.00) | 0.153 |
| Sex (%), female | 142 (64.0) | 85 (63.9) | 57 (64.0) | 1 |
| NIHSS baseline (median (IQR)) | 28.00 (20.00- 34.00) | 32.00 (25.00- 35.00) | 22.00 (12.00- 30.00) | <0.001 |
| pc-ASPECTS baseline (median (IQR)) | 8.00 (7.00- 10.00) | 7.00 (6.00- 9.00) | 9.00 (8.00- 10.00) | <0.001 |
| PC-CS Score (median (IQR)) | 5.00 (4.00- 6.00) | 4.00 (4.00- 6.00) | 5.00 (4.00- 6.00) | 0.005 |
| BATMAN Score (median (IQR)) | 4.00 (3.00- 5.00) | 4.00 (3.00- 5.00) | 4.00 (3.00- 6.00) | 0.004 |
| Intravenous Thrombolysis (%) | 45 (20.3) | 27 (20.3) | 18 (20.2) | 1 |
| Prodrome syndrome (%) | 76 (34.2) | 42 (31.6) | 34 (38.2) | 0.382 |
| SBP (mean (SD)) | 148.55 (25.31) | 150.68 (26.93) | 145.37 (22.45) | 0.126 |
| DBP (median (IQR)) | 84.00 (76.00- 96.75) | 84.00 (77.00- 99.00) | 84.00 (75.00- 93.00) | 0.258 |
| **medical history** |  |  |  |  |
| Hypertension (%) | 147 (66.2) | 91 (68.4) | 56 (62.9) | 0.481 |
| Hyperlipidemia (%) | 65 (29.3) | 39 (29.3) | 26 (29.2) | 1 |
| Diabetes mellitus (%) | 44 (19.8) | 30 (22.6) | 14 (15.7) | 0.281 |
| Smoking (%) | 69 (31.1) | 39 (29.3) | 30 (33.7) | 0.587 |
| Atrial fibrillation (%) | 104 (46.8) | 68 (51.1) | 36 (40.4) | 0.154 |
| **time intervals, min (median (IQR))** | |  |  |  |
| Onset To Imaging | 185.00 (85.00- 306.50) | 180.00 (85.00- 330.00) | 198.00 (89.00- 293.00) | 0.637 |
| Onset To Treatment | 227.00 (129.75- 357.50) | 219.00 (128.00- 378.00) | 234.00 (138.00- 329.00) | 0.648 |
| Onset To Puncture | 288.00 (197.25- 432.00) | 291.00 (204.00- 458.00) | 278.00 (193.00- 394.00) | 0.617 |
| Onset To Recanalization | 378.00 (296.75- 515.00) | 412.00 (306.50- 568.50) | 359.00 (281.00- 495.00) | 0.064 |
| Puncture To Recanalization | 87.00 (59.75- 124.50) | 102.00 (65.00- 138.50) | 73.00 (52.02- 105.00) | <0.001 |
| Successful reperfusion (%) | 188 (84.7) | 103 (77.4) | 85 (95.5) | 0.001 |
| Stroke etiology (%) |  |  |  | 0.428 |
| LAA | 65 (29.3) | 35 (26.3) | 30 (33.7) |  |
| CE | 131 (59.0) | 83 (62.4) | 48 (53.9) |  |
| Others | 26 (11.7) | 15 (11.3) | 11 (12.4) |  |

Abbreviations: IQR, interquartile; SD, standard deviation; NA, not applicable; NIHSS, National Institutes of Health Stroke Scale; pc-ASPECTS, posterior circulation–Alberta Stroke Program Early CT Score; PC–CS, posterior circulation collateral score; BATMAN, basilar artery on Tomography Angiography; SBP, systolic blood pressure; DBP, diastolic blood pressure; LAA, large artery atherosclerosis; CE, cardio embolism.

Supplemental Figure I. Study flowchart.


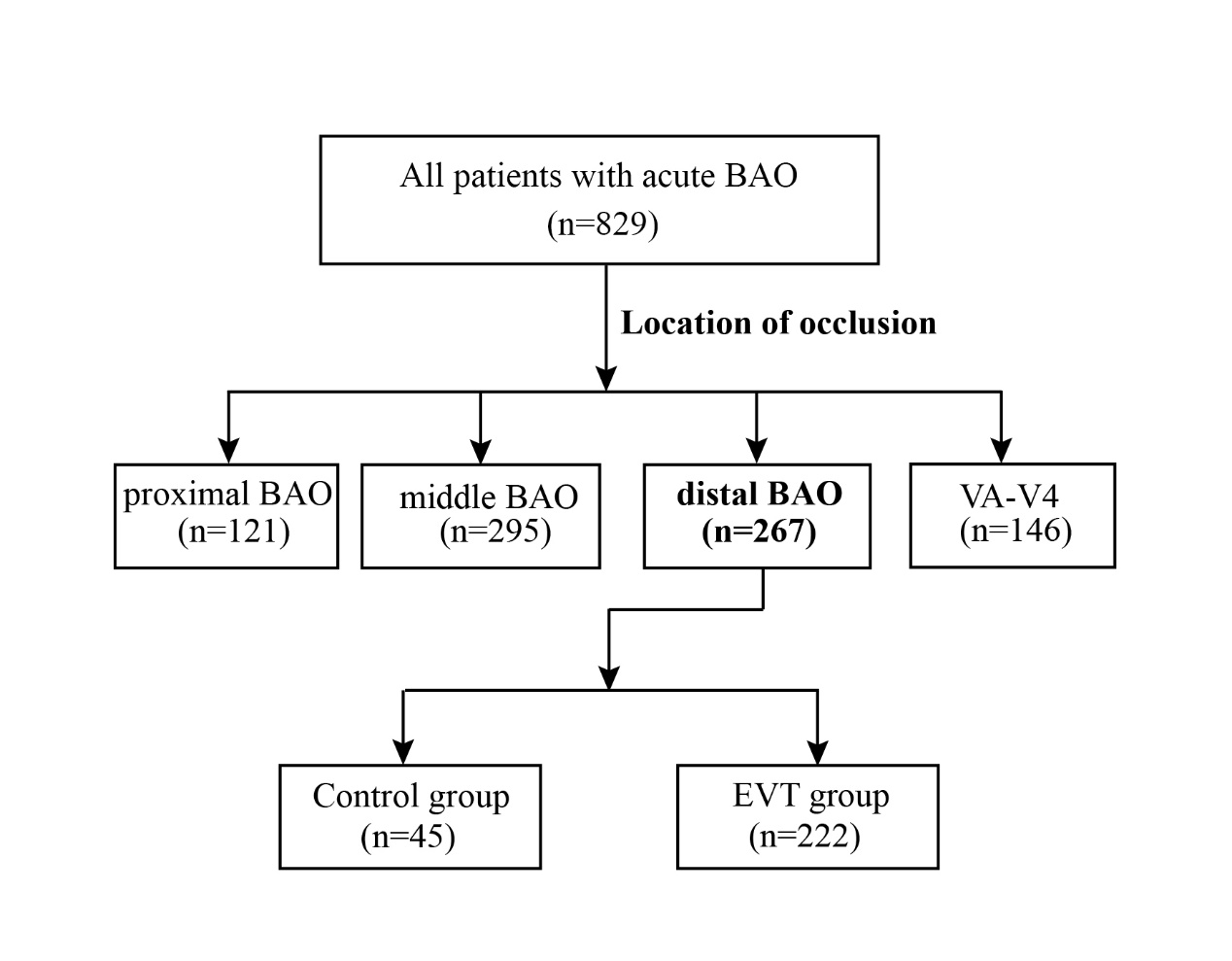

Supplement: Supplementary file 1 [file Data_Sheet_1.docx]
